# Supplementary material for: Static and dynamic coding in distinct cell types during associative learning in the prefrontal cortex
Source: Nat Commun. 2023 Dec 14;14:8325. doi: 10.1038/s41467-023-43712-2 (PMC10721651; doi:10.1038/s41467-023-43712-2)
Supplement: Supplementary file 1 — Supplementary Information [file 41467_2023_43712_MOESM1_ESM.pdf]

Supplementary Materials for

Static and dynamic coding in distinct cell types during associative learning in the prefrontal cortex

Francesco Ceccarelli, Lorenzo Ferrucci, Fabrizio Londei, Surabhi Ramawat, Emiliano Brunamonti, Aldo Genovesio*

*Corresponding author Email: aldo.genovesio@uniroma1.it (A.G.)

This PDF file includes:

Supplementary Fig. 1
Supplementary Fig. 2
Supplementary Fig. 3
Supplementary Fig. 4
Supplementary Fig. 5
Supplementary Fig. 6
Supplementary Fig. 7
Supplementary Fig. 8
Supplementary Fig. 9
Supplementary Fig. 10

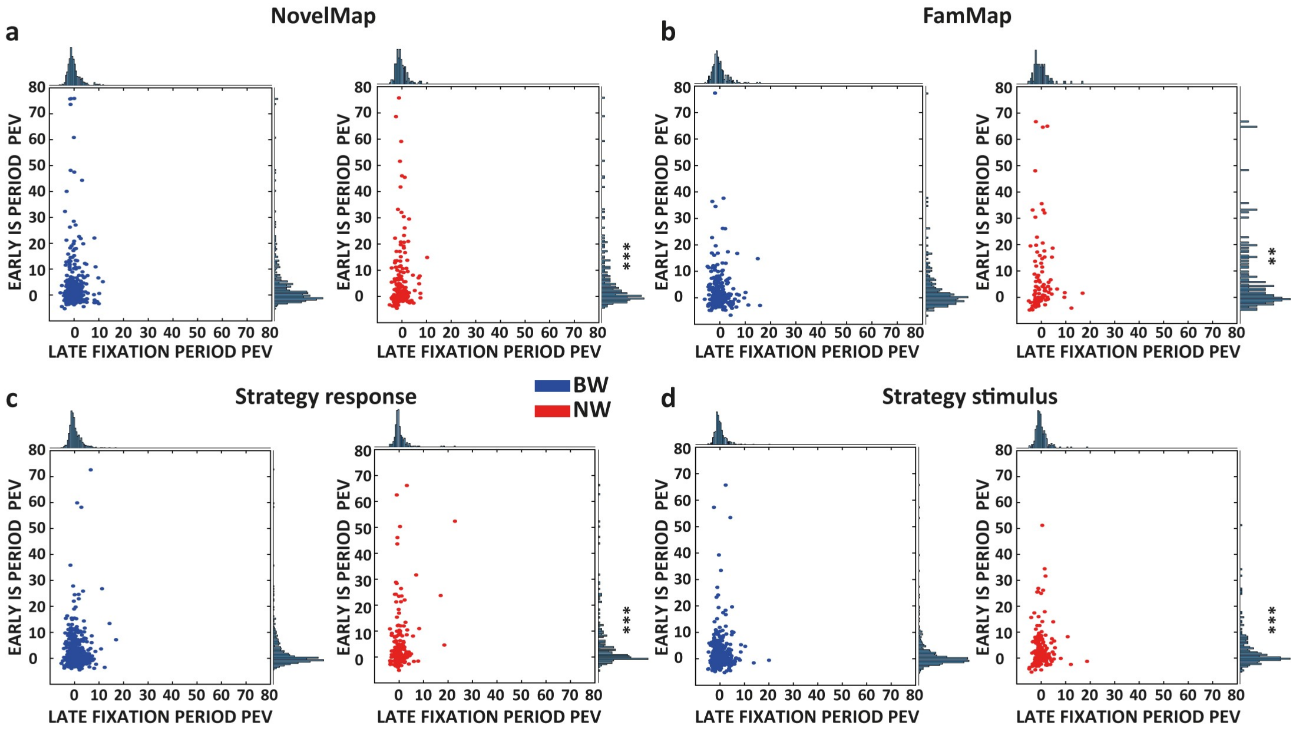

Supplementary Figure 1. Explained variance of cell type populations between the late fixation and the early IS period. Scatterplots display the explained variance values for each cell of the last 500 ms of the fixation period and the following 500 ms of the early IS period, for NovelMap (a), FamMap (b), and for response (c) and stimulus (d) in the Strategy task. Distributions in each plot represent the explained variance values in the fixation (top) and IS periods (right). Asterisks represent a significant difference between the cell encoding distributions of cell types, indicating a higher coding magnitude in the NW population. Kruskal-Wallis test: **: $p < 0.01$; ***: $p < 0.001$ (a) $p = 7.9 \times 10^{-5}$; b) $p = 0.004$; c) $p = 1.7 \times 10^{-4}$; d) $p = 1.1 \times 10^{-4}$). Source data are provided as a Source Data file.

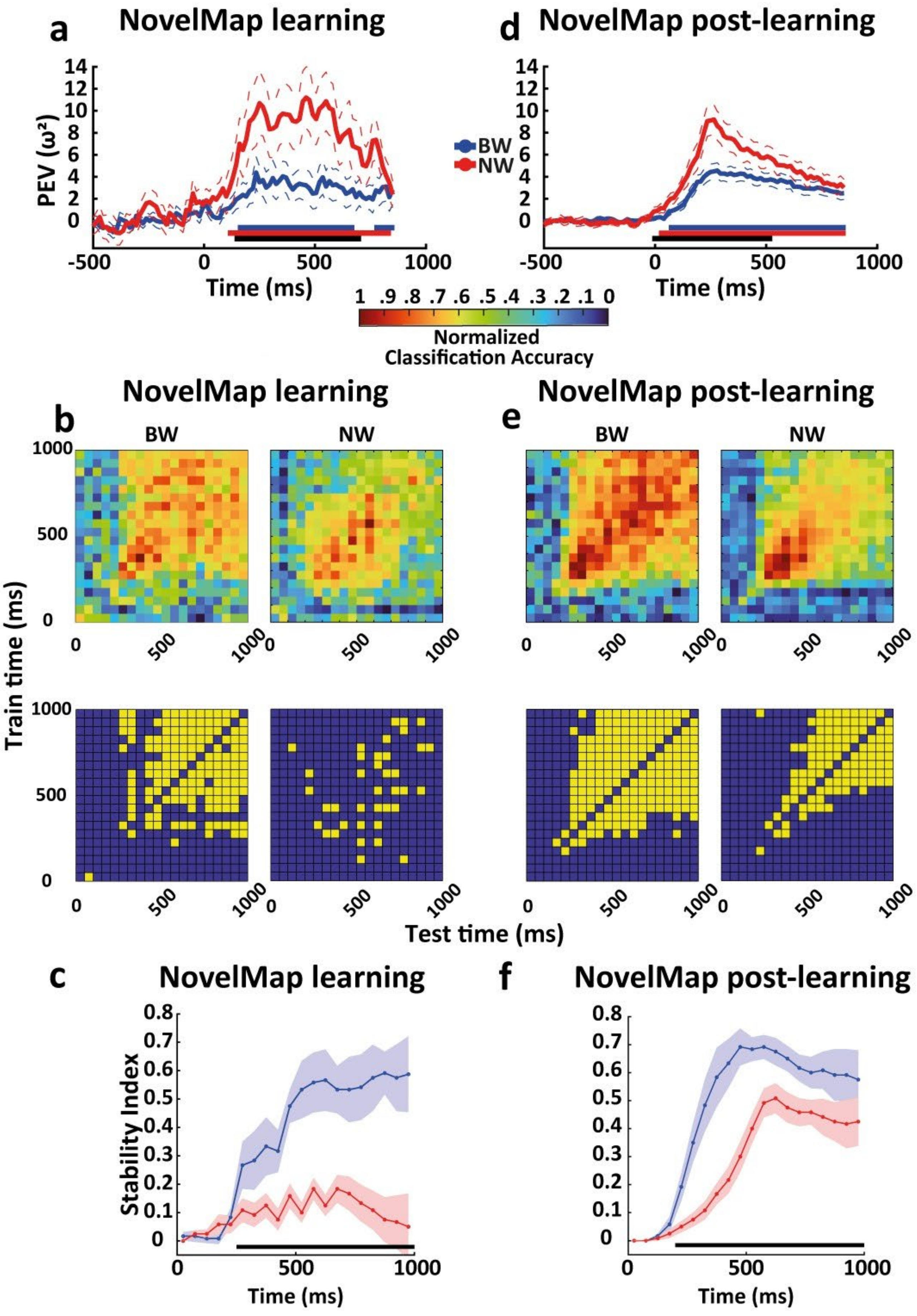

Supplementary Figure 2. Cross-temporal decoding, stable data point classification, stability index and explained variance in within-session NovelMap. Learning block of

trials (a, b, c), and post-learning block of trials (d, e, f) in the NovelMap task. The organization of the figure is the same as that in Fig. 3 and Fig. 4 for explained variance and cross-temporal decoding analysis, respectively. We applied a number-matching procedure (see Methods), selecting 40 cells for each cell type population and trial block. In the classification maps, each data point (on-diagonal points are not considered in this analysis and left in blue by convention) is in yellow if static or blue if it is not (see Methods). c, f Coding stability over time is quantified by the stability index for each cell type, where high values correspond to pronounced stability (see Methods). Shadow areas represent ±1 SD of indices computed independently by repeating decoding by resampling neurons with replacement for each cell type population (see Methods). Black bars show the time points with a significant difference in stability (cluster-based permutation test < 0.001) or ω^2 values (cluster-based permutation test < 0.001) between the two populations. Red and blue bars mark ω^2 values significantly higher than the null distribution (cluster-based permutation test $p < 0.001$, see Methods). Source data are provided as a Source Data file.

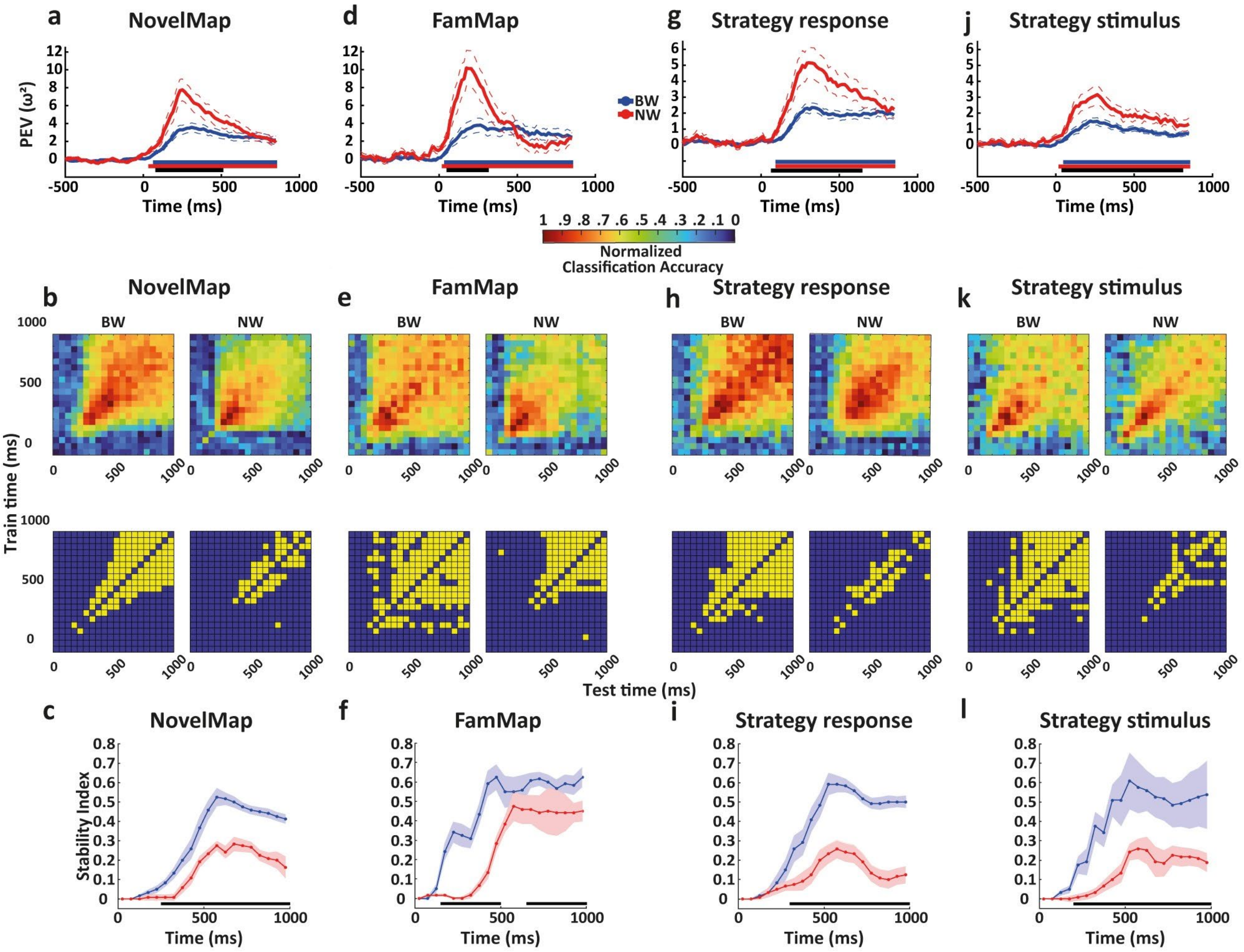

Supplementary Figure 3. Cross-temporal decoding, stable data point classification, stability index and explained variance in for populations with matched firing. Novel associations in NovelMap (a, b, c), familiar associations in FamMap (d, e, f), and response (g, h, i) and stimulus (j, k, l) in the Strategy task. The organization of the figure is the same as that in Fig. 3 and Fig. 4 for explained variance and cross-temporal decoding analysis, respectively. Black bars show the time points with a significant difference in stability (cluster-based permutation test < 0.001) or ω^2 values (cluster-based permutation test < 0.001) between the two populations. Red and blue bars mark ω^2 values significantly higher than the null distribution (cluster-based permutation test $p < 0.001$, see Methods). Shadow areas represent ±1 SD of indices computed independently by repeating decoding by resampling neurons with replacement for each cell type population (see Methods). Source data are provided as a Source Data file.

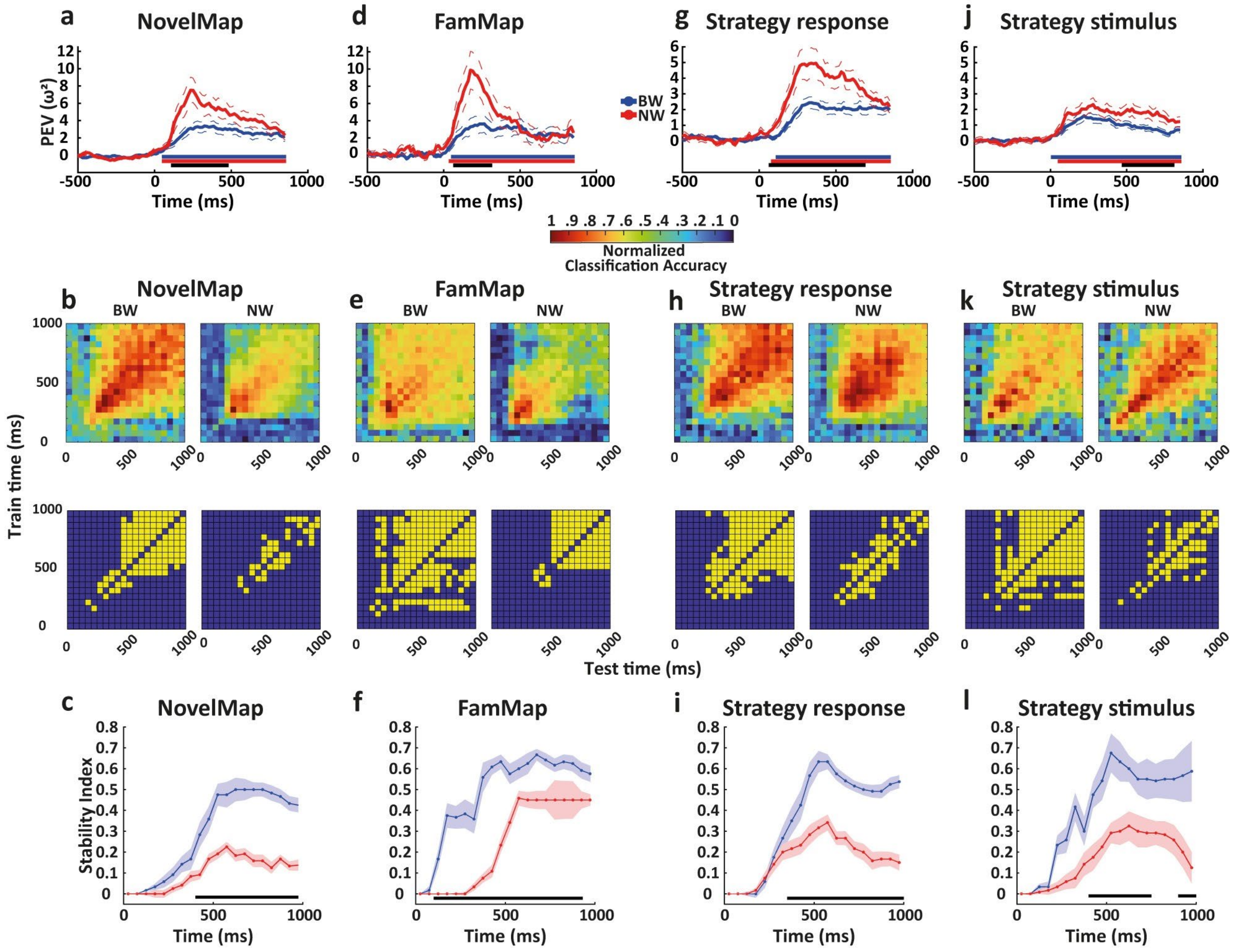

Supplementary Figure 4. Cross-temporal decoding, stable data point classification, stability index and explained variance for task-related cells. Novel associations in NovelMap (a, b, c), familiar associations in FamMap (d, e, f), and response (g, h, i) and stimulus (j, k, l) in the Strategy task. The organization of the figure is the same as that in Fig. 3 and Fig. 4 for explained variance and cross-temporal decoding analysis, respectively. Black bars show the time points with a significant difference in stability (cluster-based permutation test < 0.001) or ω^2 values (cluster-based permutation test < 0.001) between the two populations. Red and blue bars mark ω^2 values significantly higher than the null distribution (cluster-based permutation test $p < 0.001$, see Methods). Shadow areas represent ±1 SD of indices computed independently by repeating decoding by resampling neurons with replacement for each cell type population (see Methods). Source data are provided as a Source Data file.

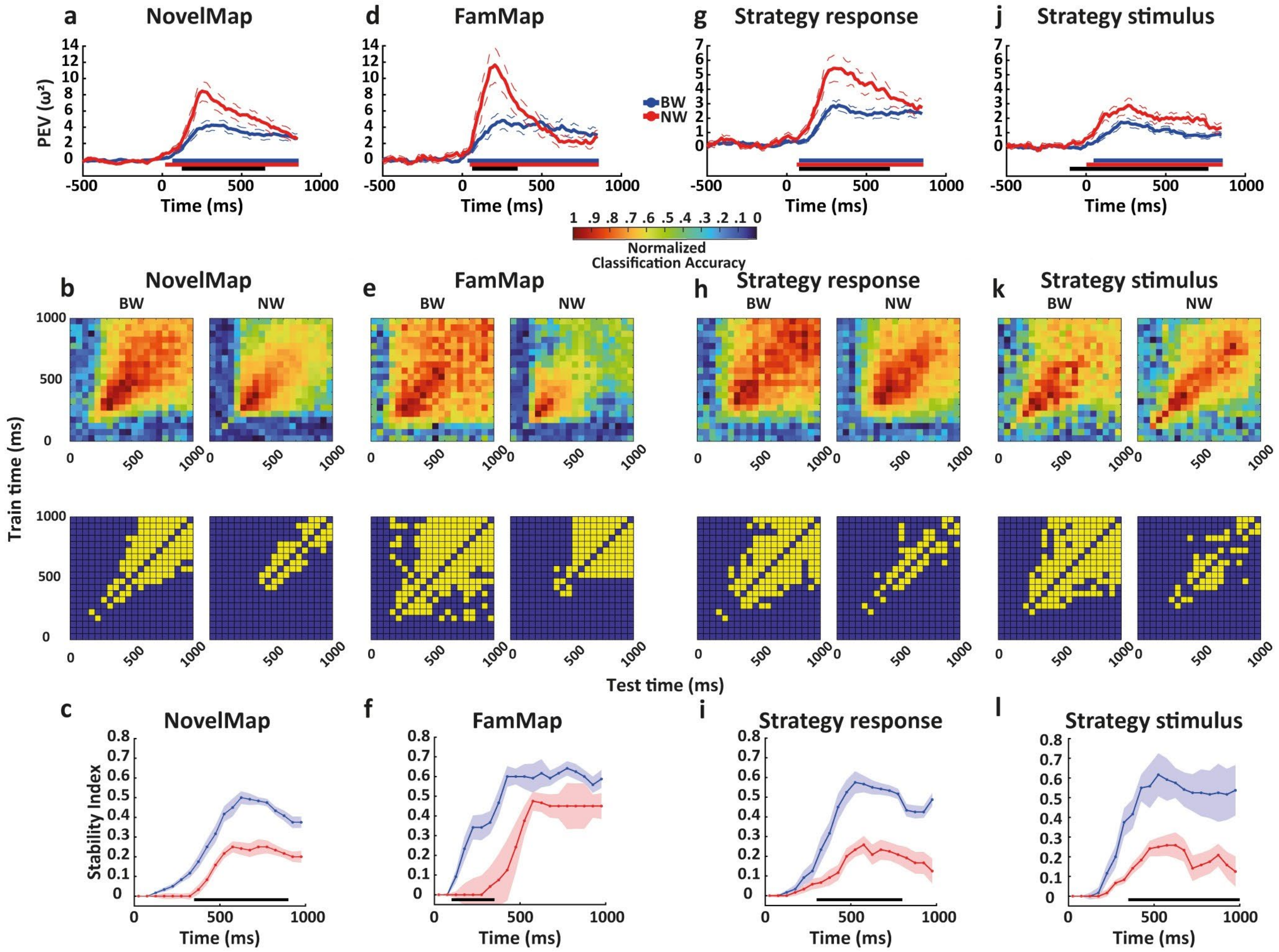

Supplementary Figure 5. Cross-temporal decoding, stable data point classification, stability index and explained variance in cell type populations with firing greater than 0.5 Hz. Novel associations in NovelMap (a, b, c), familiar associations in FamMap (d, e, f), and response (g, h, i) and stimulus (j, k, l) in the Strategy task. The organization of the figure is the same as that in Fig. 3 and Fig. 4 for explained variance and cross-temporal decoding analysis, respectively. Black bars show the time points with a significant difference in stability (cluster-based permutation test < 0.001) or ω^2 values (cluster-based permutation test < 0.001) between the two populations. Red and blue bars mark ω^2 values significantly higher than the null distribution (cluster-based permutation test $p < 0.001$, see Methods). Shadow areas represent ±1 SD of indices computed independently by repeating decoding by resampling neurons with replacement for each cell type population (see Methods). Source data are provided as a Source Data file.

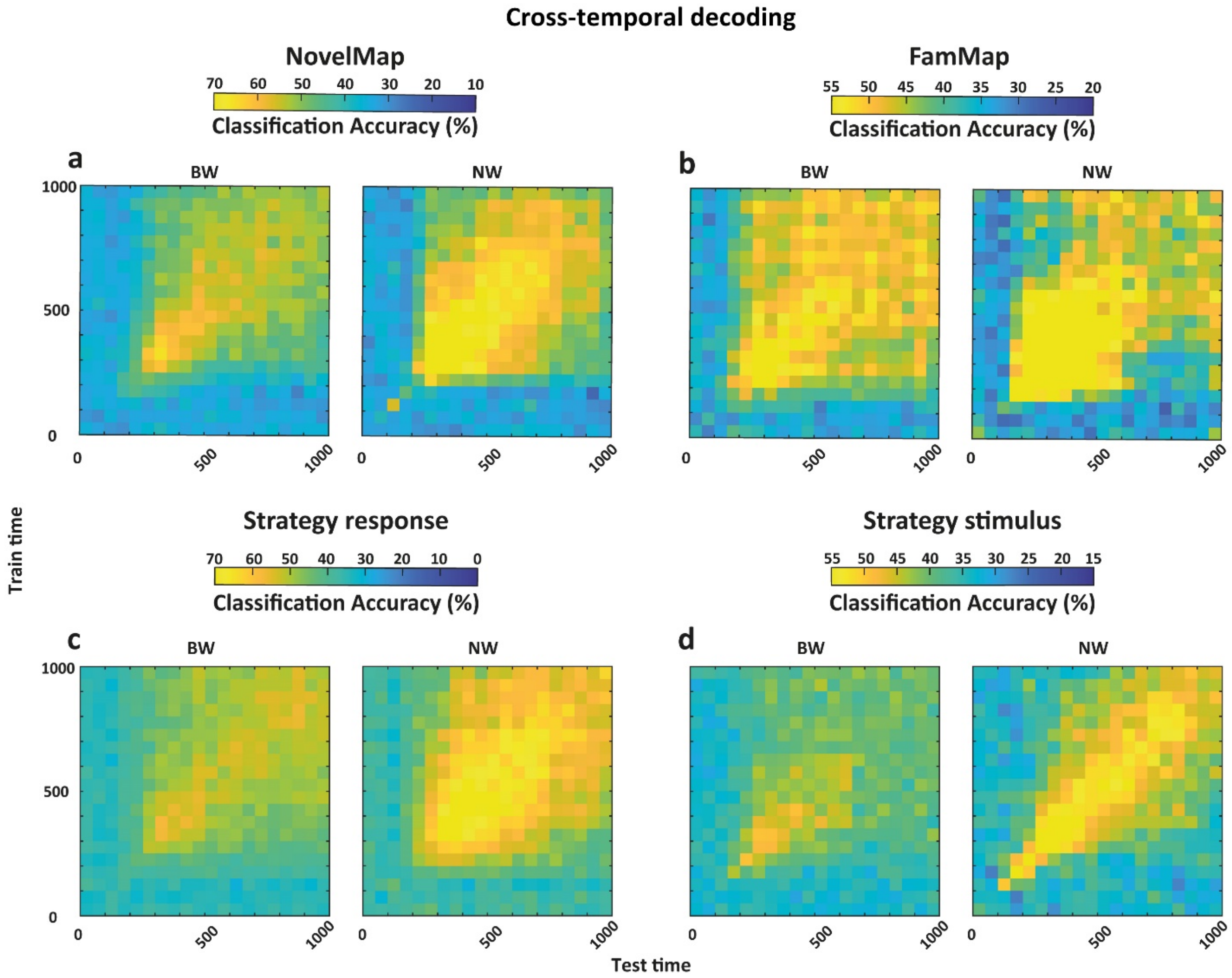

Supplementary Figure 6. Cross-temporal decoding classification accuracy. Color maps corresponding to the analysis in Fig. 4 and 5, show non-normalized classification accuracy values for (a) novel associations in NovelMap, (b) familiar associations in FamMap, and (c) response, and (d) stimulus in the Strategy task. Source data are provided as a Source Data file.

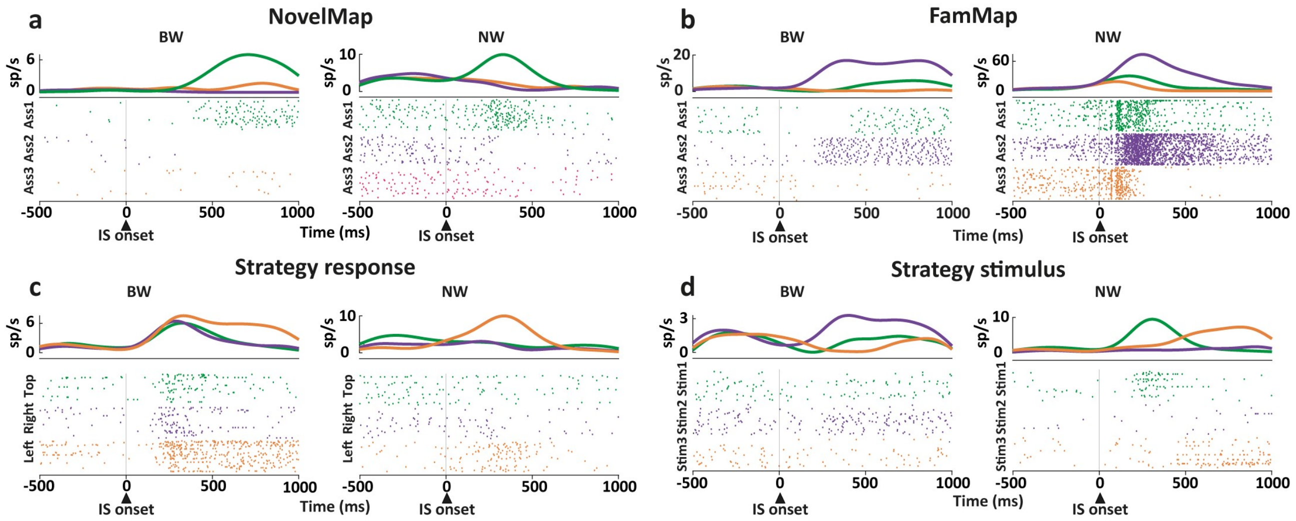

Supplementary Figure 7. Raster plots for each cell type and task. Single unit activity during late fixation and IS period for BW and NW example cells for novel associations in NovelMap (a), familiar associations in FamMap (b), response (c), and stimulus (d) in the Strategy task. The black triangle shows alignment at the IS onset. Colored lines (top part of each raster) show spike density activity for each condition of the variable. Source data are provided as a Source Data file.

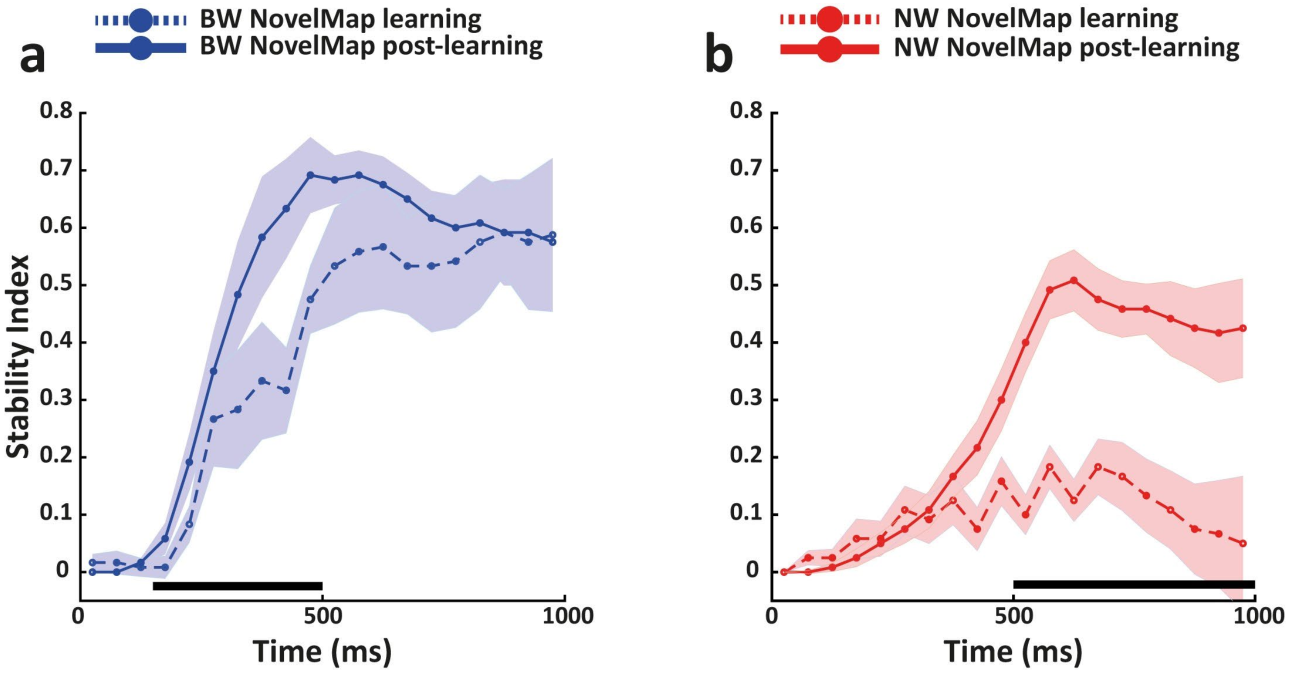

Supplementary Figure 8. Stability index comparison between within-session NovelMap trial blocks for cell types. Learning block of trials (dashed lines) and post-learning block of trials in the NovelMap task (solid lines) are directly compared for the BW (a) and NW (b) populations, respectively. Black bars indicate a significant difference in the stability levels between the two tasks (cluster-based permutation test < 0.001). Shadow areas represent ±1 SD of indices computed independently by repeating decoding by resampling neurons with replacement for each cell type population (see Methods). Source data are provided as a Source Data file.

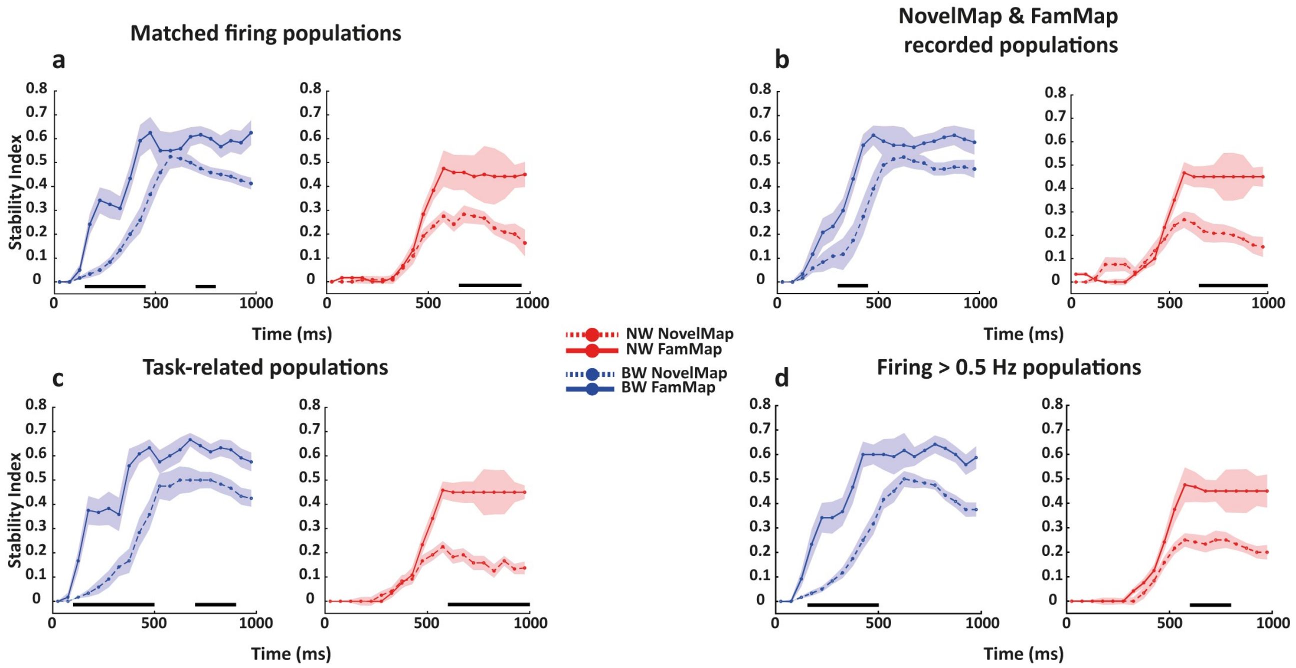

Supplementary Figure 9. Stability index comparison between associative tasks for control analysis. Novel associations in NovelMap (dashed lines) and familiar associations in the FamMap (solid lines) tasks are directly compared for both the cell types and different populations characterized by matched firing (a), recording in both associative tasks (b), task-related activity (c), cut off firing >= 0.5 Hz (d). Black bars indicate a significant difference in the stability levels between the two tasks (cluster-based permutation test < 0.001). Shadow areas represent ±1 SD of indices computed independently by repeating decoding by resampling neurons with replacement for each cell type population (see Methods). Source data are provided as a Source Data file.

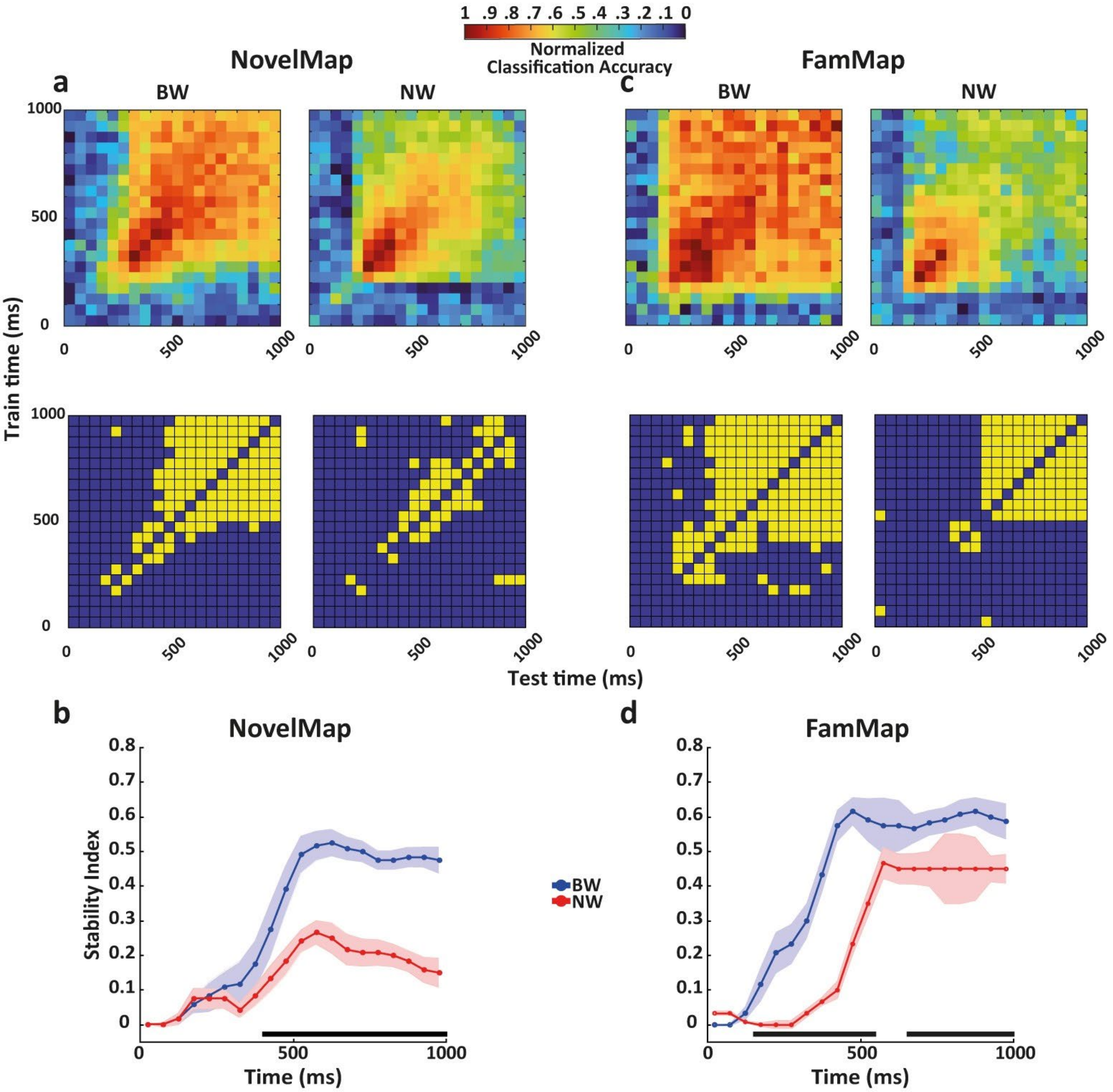

Supplementary Figure 10. Cross-temporal decoding, stable data point classification, stability index in cells recorded in the same daily session. Novel associations in NovelMap (a, b), familiar associations in FamMap (c, d). The organization of the figure is the same as that in Fig. 4. Horizontal black lines show the time points with a significant difference in stability between the two populations (cluster-based permutation test $p < 0.001$). Shadow areas represent ±1 SD of indices computed independently by repeating decoding by resampling neurons with replacement for each cell type population (see Methods). Source data are provided as a Source Data file.
