# Supplementary material for: Static and dynamic coding in distinct cell types during associative learning in the prefrontal cortex
Source: Nat Commun. 2023 Dec 14;14:8325. doi: 10.1038/s41467-023-43712-2 (PMC10721651; doi:10.1038/s41467-023-43712-2)
Supplement: Supplementary file 3 — Reporting Summary [file 41467_2023_43712_MOESM3_ESM.pdf]

nature portfolio

Corresponding author(s): Aldo Genovesio

Last updated by author(s): 30/10/2023

Reporting Summary

Nature Portfolio wishes to improve the reproducibility of the work that we publish. This form provides structure for consistency and transparency in reporting. For further information on Nature Portfolio policies, see our Editorial Policies and the Editorial Policy Checklist.

Statistics

For all statistical analyses, confirm that the following items are present in the figure legend, table legend, main text, or Methods section.

n/a	Confirmed	
☐	☒	The exact sample size (n) for each experimental group/condition, given as a discrete number and unit of measurement
☐	☒	A statement on whether measurements were taken from distinct samples or whether the same sample was measured repeatedly
☐	☒	The statistical test(s) used AND whether they are one- or two-sided<br>*Only common tests should be described solely by name; describe more complex techniques in the Methods section.*
☒	☐	A description of all covariates tested
☐	☒	A description of any assumptions or corrections, such as tests of normality and adjustment for multiple comparisons
☐	☒	A full description of the statistical parameters including central tendency (e.g. means) or other basic estimates (e.g. regression coefficient) AND variation (e.g. standard deviation) or associated estimates of uncertainty (e.g. confidence intervals)
☐	☒	For null hypothesis testing, the test statistic (e.g. F, t, r) with confidence intervals, effect sizes, degrees of freedom and P value noted<br>*Give P values as exact values whenever suitable.*
☒	☐	For Bayesian analysis, information on the choice of priors and Markov chain Monte Carlo settings
☒	☐	For hierarchical and complex designs, identification of the appropriate level for tests and full reporting of outcomes
☒	☐	Estimates of effect sizes (e.g. Cohen's d, Pearson's r), indicating how they were calculated

Our web collection on statistics for biologists contains articles on many of the points above.

Software and code

Policy information about availability of computer code

Data collection	NIHM CORTEX was used to collect behavioural data (software can be found at https://www.nimh.nih.gov/research/research-conducted-at-nimh/research-areas/clinics-and-labs/ln/shn/software-projects). Electrophysiological data were collected with Plexon Multichannel Acquisition Processor or Alpha-Omega Multispike Detector.
Data analysis	Cell types were classified using The Waveform Analysis toolbox (https://bitbucket.org/sardid/waveformanalysis/src/master/). Analysis of explained variance was calculated using The Measures of Effect Size (MES) Toolbox (https://github.com/hhentschke/measures-of-effect-size-toolbox) and decoding analysis with The Neural Decoding Toolbox (http://www.readout.info/). Custom code used to classify static data points and calculate stability index is available at https://osf.io/bwnq9/. Data were analyzed using MATLAB 2021b.

For manuscripts utilizing custom algorithms or software that are central to the research but not yet described in published literature, software must be made available to editors and reviewers. We strongly encourage code deposition in a community repository (e.g. GitHub). See the Nature Portfolio guidelines for submitting code & software for further information.

Data

Policy information about availability of data

All manuscripts must include a data availability statement. This statement should provide the following information, where applicable:

- Accession codes, unique identifiers, or web links for publicly available datasets
- A description of any restrictions on data availability
- For clinical datasets or third party data, please ensure that the statement adheres to our policy

The data necessary for the evaluation of this study are provided with the data source file and the additional data provided in the https://osf.io/bwnq9/ repository. Raw data are available on request from the corresponding author. Source data are provided with this paper.

Research involving human participants, their data, or biological material

Policy information about studies with human participants or human data. See also policy information about sex, gender (identity/presentation), and sexual orientation and race, ethnicity and racism.

Reporting on sex and gender	N/A
Reporting on race, ethnicity, or other socially relevant groupings	N/A
Population characteristics	N/A
Recruitment	N/A
Ethics oversight	N/A

Note that full information on the approval of the study protocol must also be provided in the manuscript.

Field-specific reporting

Please select the one below that is the best fit for your research. If you are not sure, read the appropriate sections before making your selection.

☒ Life sciences ☐ Behavioural & social sciences ☐ Ecological, evolutionary & environmental sciences

For a reference copy of the document with all sections, see nature.com/documents/nr-reporting-summary-flat.pdf

Life sciences study design

All studies must disclose on these points even when the disclosure is negative.

Sample size	No power analysis was performed before the beginning of the study. In non-human primates experiments, ethical and practical constrains typically limit the sample size to two animals.
Data exclusions	For the analyses in Figure 2, all the data composed of well-isolated single cells were used. All subsequent analyses included cells with at least 10 trials recorded for the variables of interest, as explicitly reported in the Results section. Finally, the Figures reported in the Supplementary file included only cells with comparable firing rates (Supplementary Figures 3), task-related (Supplementary Figures 4), firing greater than 0.5 Hz (Supplementary Figures 5) and recorded in both associative tasks (Supplementary Figures10), as described in the Results section.
Replication	The animals performed the experimental tasks reported in this study across numerous recording days (Approximately 103 days of recording, with several specific recording blocks for each task studied each day). The main results were replicateci using a subset of data balanced by firing rate and cells utilized, analyses explicitly described in the results section and reported in the Supplementary file.
Randomization	Trials of the experiment were balanced and randomized between variables of interest.
Blinding	In non-human primates experiments, practical constrains related to animal management do not allow blinding.

Reporting for specific materials, systems and methods

We require information from authors about some types of materials, experimental systems and methods used in many studies. Here, indicate whether each material, system or method listed is relevant to your study. If you are not sure if a list item applies to your research, read the appropriate section before selecting a response.

Materials & experimental systems

n/a	Involved in the study
☒	☐ Antibodies
☒	☐ Eukaryotic cell lines
☒	☐ Palaeontology and archaeology
☐	☒ Animals and other organisms
☒	☐ Clinical data
☒	☐ Dual use research of concern
☒	☐ Plants

Methods

n/a	Involved in the study
☒	☐ ChIP-seq
☒	☐ Flow cytometry
☒	☐ MRI-based neuroimaging

Animals and other research organisms

Policy information about studies involving animals; ARRIVE guidelines recommended for reporting animal research, and Sex and Gender in Research

Laboratory animals	Two adult Rhesus macaques (Macaca mulatta).
Wild animals	This study did not involve wild animail
Reporting on sex	Two adult male Rhesus macaques (Macaca mulatta) were used for this study.
Field-collected samples	This study did not involve samples collected in the field.
Ethics oversight	All procedures conformed to the Guide for the Care and Use of Laboratory Animals (1996) and were approved by the National Institute of Mental Health Animai Care and Use Committee.

Note that full information on the approval of the study protocol must also be provided in the manuscript.

Plants

Seed stocks	N/A
Novel plant genotypes	N/A
Authentication	N/A
